# Exome and deep sequencing of clinically aggressive neuroblastoma reveal somatic mutations that affect key pathways involved in cancer progression

**DOI:** 10.18632/oncotarget.8187

**Published:** 2016-03-18

**Authors:** Vito Alessandro Lasorsa, Daniela Formicola, Piero Pignataro, Flora Cimmino, Francesco Maria Calabrese, Jaume Mora, Maria Rosaria Esposito, Marcella Pantile, Carlo Zanon, Marilena De Mariano, Luca Longo, Michael D. Hogarty, Carmen de Torres, Gian Paolo Tonini, Achille Iolascon, Mario Capasso

**Affiliations:** ^1^ University of Naples Federico II, Department of Molecular Medicine and Medical Biotechnology, Naples, Italy; ^2^ CEINGE Biotecnolgie Avanzate, Naples, Italy; ^3^ University of Bari, Department of Biology, Bari, Italy; ^4^ Hospital Sant Joan de Déu, Developmental Tumor Biology Laboratory and Department of Oncology, Esplugues de Llobregat, Barcelona, Spain; ^5^ Pediatric Research Institute (IRP), Fondazione Città della Speranza, Neuroblastoma Laboratory, Padua, Italy; ^6^ U.O.C. Bioterapie, IRCCS AOU San Martino-IST, National Cancer Research Institute, Genoa, Italy; ^7^ Children's Hospital of Philadelphia, Division of Oncology, Department of Pediatrics, Perelman School of Medicine at the University of Pennsylvania, Philadelphia, PA, United States of America; ^8^ IRCCS SDN, Istituto di Ricerca Diagnostica e Nucleare, Naples, Italy

**Keywords:** NGS, neuroblastoma, high risk, somatic mutation, cancer driver genes

## Abstract

The spectrum of somatic mutation of the most aggressive forms of neuroblastoma is not completely determined. We sought to identify potential cancer drivers in clinically aggressive neuroblastoma.

Whole exome sequencing was conducted on 17 germline and tumor DNA samples from high-risk patients with adverse events within 36 months from diagnosis (HR-Event3) to identify somatic mutations and deep targeted sequencing of 134 genes selected from the initial screening in additional 48 germline and tumor pairs (62.5% HR-Event3 and high-risk patients), 17 HR-Event3 tumors and 17 human-derived neuroblastoma cell lines.

We revealed 22 significantly mutated genes, many of which implicated in cancer progression. Fifteen genes (68.2%) were highly expressed in neuroblastoma supporting their involvement in the disease. *CHD9*, a cancer driver gene, was the most significantly altered (4.0% of cases) after *ALK*.

Other genes (*PTK2*, *NAV3*, *NAV1*, *FZD1* and *ATRX*), expressed in neuroblastoma and involved in cell invasion and migration were mutated at frequency ranged from 4% to 2%.

Focal adhesion and regulation of actin cytoskeleton pathways, were frequently disrupted (14.1% of cases) thus suggesting potential novel therapeutic strategies to prevent disease progression.

Notably *BARD1*, *CHEK2* and *AXIN2* were enriched in rare, potentially pathogenic, germline variants.

In summary, whole exome and deep targeted sequencing identified novel cancer genes of clinically aggressive neuroblastoma. Our analyses show pathway-level implications of infrequently mutated genes in leading neuroblastoma progression.

## INTRODUCTION

Neuroblastoma is a pediatric tumor resulting from malignant transformation of neural crest-derived precursors of the peripheral sympathetic nervous system. Although many low and intermediate-risk patients are amenable to no or little therapy, overall survival rates of patients diagnosed as high-risk tumors remain below 50% despite receiving complex and intensive treatments [1]. Among high-risk patients, gene signatures can identify children with higher risk disease who would benefit from new and more aggressive therapeutic approaches [2]. Genome-wide association studies have identified multiple DNA polymorphisms influencing neuroblastoma susceptibility and clinical phenotype [3-7]. High-throughput sequencing-based studies have highlighted that recurrent mutations of single genes are infrequent in primary neuroblastoma with activating mutations in *ALK* and inactivating mutations in *ATRX,* and *TERT* rearrangements being the most frequent [8-11]. Two recent studies have shown a higher mutational load in relapsed neuroblastoma than in primary tumors with a relevant proportion of mutations in genes of the RAS-MAPK and YAP pathways [12, 13]. Nevertheless, genes infrequently mutated but acting in molecular mechanisms underlying the oncogenesis and progression of neuroblastoma remain unknown, and targeted therapies identified to date, such as ALK inhibitors, might benefit a reduced number of patients [14]. It is thought that rarely mutated genes may also contribute to tumor development, thus accounting for inter-tumor variability. Recent studies have painted a portrait of the mutation landscape for multiple cancers including pancreatic, lung, breast, brain and ovarian. In each case, the distribution of somatic point mutations across the samples typically includes a few altered genes at frequencies higher than 10% and a long “tail” of many genes mutated at frequencies of 5% or lower [15]. Driver genes are detected mostly from positive-selection signals found in the mutation patterns of individual genes across tumors [15]. However, this approach will miss less-frequently mutated but functionally important genes that a typical cohort with hundreds of tumor samples is not statistically powered to detect.

Here we used a strategy to identify somatic mutations that are rare at the gene level, but frequently affect specific processes of biological relevance in aggressive neuroblastoma. Moreover, we sought to identify potential variants predisposing to aggressive neuroblastoma.

## RESULTS

### Whole exome (WES) and deep targeted (DT-seq) sequencing

**Figure 1:**
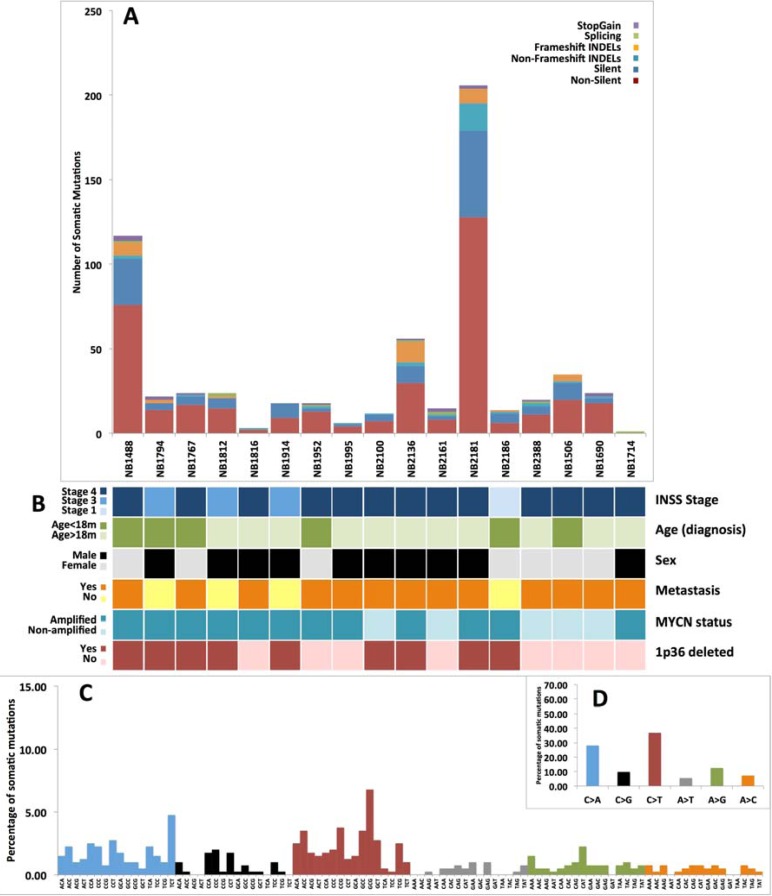
Somatic variants annotation and somatic signature **A.** The top bar plot shows the functional classes frequencies for the annotated variants in each case of tumor. **B.** The data grid summarize clinical information of neuroblastoma samples. **C.** The bottom histogram shows the detailed spectrum of somatic SNVs. The Y axis reports the frequency of nucleotide substitutions, while X axis shows the trinucleotides context in which the somatic changes occur. **D.** The nested top right box indicates the frequency of the six types of base substitutions caused by somatic mutations.

We performed WES of 17 matched germline and HR-Event3 neuroblastoma tissue pairs (Supplementary Figure 1, Supplementary Table 1 and 2a). The low rate of somatic mutations was in substantial agreement with previous studies [8-10] (Supplementary Table 2c). After stringent filtering steps we obtained a total of 444 non-silent somatic changes (median per sample: 17) (Figure 1A and 1B and Supplementary Table 3a). Neuroblastoma mutation spectrum (Figure 1C and 1D) was enriched in C > A transversions (28.07%) and C > T transitions (36.84%), a process attributed to the normal cellular event of deamination of 5-methylcytosine. Our results were consistent with that reported in Alexandrov et al. [16] for neuroblastoma that is characterized by two signatures: C > T changes (signature 1b, common and widespread among diverse cancer types) that contribute to the 53.2%, and C > A variations (signature 18, a finding considered to be unique for neuroblastoma) that contribute to the 46.8% of the overall pattern. We also compared our data to those published in previous reports [8-10] (Supplementary Figure 2) and confirmed the overall concordance of the mutational spectrum except for the C > A transversions percentage that was lower than that of C > T transitions. This could be due to the high inter-sample variability observed among datasets for the C > A mutation respect to the C > T mutation. However, the specific percentage of C > A mutations in TCT contexts and C > T in GCG triplets resulted to be consistent among the four datasets. Indeed, these were the most frequent changes observed in 3 out of 4 datasets.

### Mutated genes prioritization

To determine mutational events contributing to HR-Event3 neuroblastoma (driver events), we first selected candidate drivers from the discovery cohort obtaining 134 genes (see Supplementary information and Supplementary Table 3a). Next, we deep sequenced all exons of the 134 selected genes in an independent validation set of 48 matched tumor-control pairs (17 HR-Event3, 13 high-risk, 10 intermediate-risk, 8 low-risk), 17 HR-Event3 tumors and 17 cell lines with a mean depth of 733x (Supplementary Table 1 and 2b). Ninety-one non-silent somatic changes were identified in 55 genes (Supplementary Table 3b).

**Figure 2:**
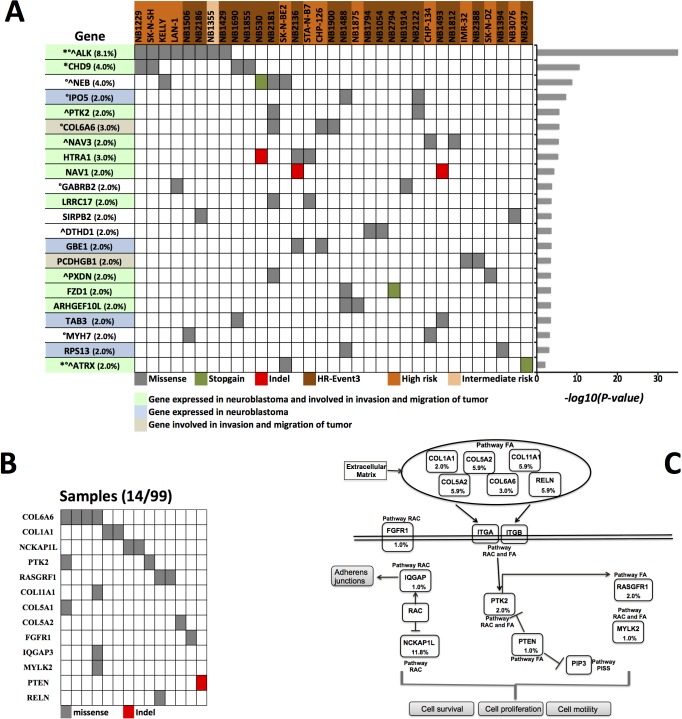
Prioritized genes and altered pathways **A.** Data matrix showing significantly mutated genes (FDR < 0.25 calculated with CHASM and FDR < 0.1 calculated with VEST) discovered in cases with clinically aggressive neuroblastoma by WES and DT-seq. The significance levels of the gene are plotted on the right. *Cancer Gene Census. Mouse insertional mutagenesis experiments support *CHD9* as cancer causing gene. °Gene found mutated with non-silent mutations in previous studies on primary neuroblastoma [8-11]. ^Gene found mutated with non-silent mutations in previous studies on relapsed neuroblastoma [12, 13]. In parenthesis the frequency of cancer driver mutations calculated on 99 samples. **B.** Somatic mutations of the focal adhesion (FA) and regulation of actin cytoskeleton (RAC) pathways. Shown is the mutation status of the genes of the FA and RAC pathway in the 14 neuroblastomas (12 HR-Event3 and 2 high-risk) that carry at least 1 non-silent mutation. **C.** The key genes of the FA and RAC pathways with mutation frequencies in neuroblastoma are shown (Fig. adapted from KEGG pathway database). The frequency for *COL11A1, COL5A1, COL5A2, IQGAP3, RELN, NCKAP1L* were estimated from 17 samples analyzed by WES. The frequencies for *COL6A6, COL1A1, FGFR1, PTEN, PTK2, RASGRF1, MYLK2* are from 99 samples analyzed by WES and DT-seq. PISS: Phosphatidylinositol signaling system.

We discovered 22 significantly mutated genes in 33 tumors through combining putative driver mutations from both cohorts and reapplying the cancer driver analysis (Figure 2A, Supplementary Table 3c). These genes were mutated in 20/33 (60%) HR-Event3, 12/33 (36%) high-risk and 1/33 (0.03%) intermediate-risk neuroblastomas. In keeping with literature, point mutations of *ALK* and *ATRX* were found in 8.1% and 1.1% of cases, respectively. Expression microarrays revealed that 15 genes (68.2%) were highly expressed in neuroblastoma supporting the biological rationale for their neuroblastoma involvement (Supplementary Figure 3A and 3B). Furthermore, an analysis encompassing 491 cancer types identified *ALK* and *CHD9* as highly expressed in neuroblastoma (Supplementary Figure 3C and 3D). The gene expression of 20 out of 22 genes was significantly different among low-risk, high-risk and HR-Event3 tumors (Supplementary Figure 4).

**Figure 3:**
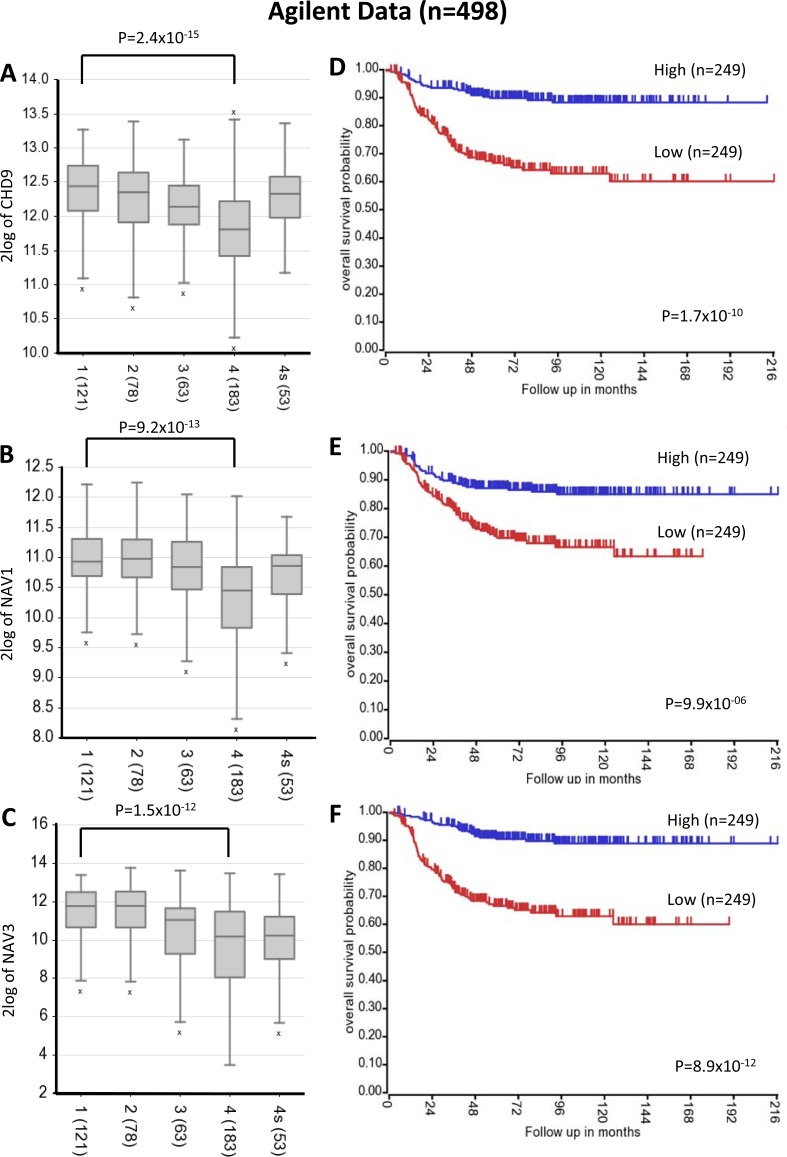
*CHD9, NAV1* and *NAV3* expression levels and survival rates Low *CHD9, NAV1* and *NAV3* expression is associated with negative prognosis and metastatic neuroblastoma stage. **A.-B.-C.** Changes in expression for *CHD9, NAV1* and *NAV3* respectively, in advanced-stage neuroblastoma using published array data (R2 bioinformatics tool). Data are shown for International Neuroblastoma Staging System stages 1-4 and 4s. The number of tumors is indicated in parentheses. **D.-E.-F.** Kaplan-Meier analysis is shown, with individuals grouped by median of expression of *CHD9, NAV1* and *NAV3,* respectively. Log-rank P values are shown.

**Figure 4:**
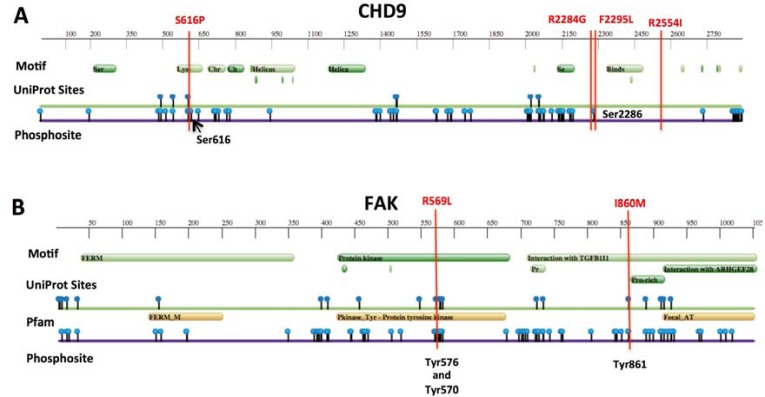
Protein feature view of Protein Data Bank entries mapped to UniProtKB sequences **A.** Graphical summary of *CHD9* and **B.** FAK (*PTK2* gene) full-length protein sequences. Vertical bars (in red) show somatic mutations found in neuroblastoma. Phosphorylation sites in Serines (in *CHD9*) or Tyrosines (in FAK) are indicated.

**Figure 5:**
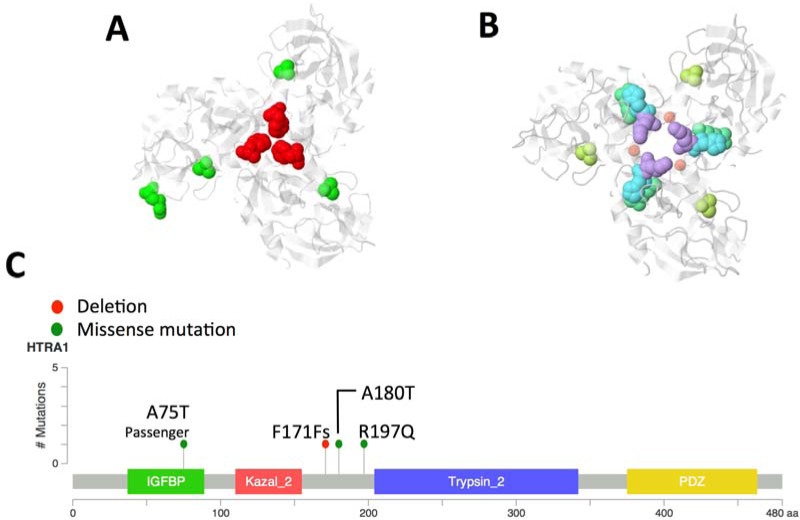
*HTRA1* cancer driver mutations Two neuroblastoma tumors (one HR-Event3 and one high-risk) carried putative driver mutations (A180T and one F171Fs) in exon 2 whereas one cell line presented a driver mutation (R197Q) in exon 3. Another non-silent mutation (A75T) was found in an intermediated risk tumor but it was not predicted to be cancer driver. All putative driver mutations are predicted to affect protein stability; particularly, F171Fs is involved in the trimer stabilization domain whereas Alanine-to-Threonine (A180T) substitution is relevant given the inverse preferences of alanine to form helices and of threonine to support beta-sheet structures. The third cancer driver mutation R197Q in exon 3 affects the binding site between two beta-sheets and is located closed to the phosphorylation site S195 (PhosphoSitePlus website). **A.** 3D model of the HTRA1 trimer. Mutations found in this study are mapped on HTRA1 protein model (PDB: 3NWU, chain A; in white); in red is shown the deletion at amino acid position 171, missense variants are colored in green. **B.** HTRA1 model is used to map mutated sites reported in The Cancer Genome Atlas (TGCA). Light purple: Liver hepatocellular carcinoma (positions 168, 180); Yellow-Green: Skin cutaneous melanoma (position 180); Light green: pancreatic adenocarcinoma (position 166); Light blue: Lung adenocarcinoma (position 167). Red dots locate the trimer stabilization portion of the protein. **C.** Mutations are mapped on the HTRA1 linearized protein model.

Four driver mutations were found in *CHD9* gene encoding for an ATP-dependent chromatin remodeling protein that induces osteogenic differentiation of mesenchymal cells [17]. We speculate that *CHD9* inactivation may increase metastatic spread to the bone, which is the second most common site of metastasis in neuroblastoma. Indeed, the osteogenesis inhibition favours bone invasion by neuroblastoma cells [18] and here we show that the downregulation of *CHD9* is correlated with metastasization and low survival rates (Figure 3A and 3D and Supplementary Figure 5A and 5D). Moreover, 3 out of 4 mutations are localized close to phosphorylation sites and thus could inhibit CHD9 protein activation (Figure 4A). Together, these data suggest that *CHD9* mutations are relevant for neuroblastoma progression. Interestingly, we found two driver mutations in *PTK2*, which encodes a focal adhesion kinase (FAK) protein, important for neuroblastoma tumor cell viability [19]. Both mutations were close to the codons for the Tyr576 and Tyr861 phosphorylation sites that are required for FAK activation (Figure 4B). Combined and concurrent inhibition of the FAK-Src-Paxillin system has been proposed as a therapy to inhibit neuroblastoma tumor growth and metastases [20]. We also focused on the serine-protease *HTRA1* since its low expression levels strongly correlate with neuroblastoma progression [21]. We found four non-silent variants, three of them predicted to be driver mutations. Two mutations occurred in exon 2 (A180T and F171Fs). Interestingly, mutation A180T has been described in other cancers (The Cancer Genome Atlas database). Since all mutations are predicted to affect HTRA1 protein stability (Figure 5), we suggest that the observed variants may act as loss-of-function mutations that enhance invasion or metastasis of tumor cells. Four mutations were found in two functionally similar genes *NAV1* and *NAV3* involved in neuronal development and cell migration [22]. Of note *NAV3* deletions have been associated with poor prognosis in nervous system tumors including neuroblastoma [22]. Accordingly, we found that downregulation of both genes is correlated with metastatic neuroblastoma and low survival rates (Figure 3B, 3C, 3D and 3F and Supplementary Figure 5B, 5C, 5D and 5F). Other five genes (*LRRC17*, *PXDN*, *FZD1*, *ARHGEF10L*, *ATRX*) resulted to be expressed in neuroblastoma and involved in cell migration and invasion (Figure 2A). Except for *ALK*, each gene was mutated at frequency ranged 4% to 2%.

### Mutational pathway enrichment analysis

We searched for enrichment of somatically mutated genes in the curated KEGG pathways through GSEA algorithm combined with mutational analysis. Focal adhesions (FA) and regulation of actin cytoskeleton (RAC) pathways were identified as the most significantly altered (FDR≤0.05; Supplementary Table 4a). Analysis of mutations from three previous reports [8-10] using our methods also identified FA and RAC among the most significantly mutated pathways (Supplementary Table 4b,c,d). Furthermore, analysis of microarray data of 498 neuroblastomas showed that FA and other pathways, implicated in cancer progression, were over-represented among over-expressed genes in low-risk tumors when compared to HR-Event3 neuroblastoma (Supplementary Table 5). Combining data from WES and DT-seq, encompassing a total of 99 samples, we found that 14.1% (14/99) of neuroblastomas (12 HR-Event3 and 2 high-risk) harbored mutations in FA and RAC pathways. In total, we detected 20 non-silent somatic mutations in 13 genes of these two pathways (Figure 2B and 2C). Most mutations in the pathways were mutually exclusive. Among cancer driver genes (Figure 2A), 13 have been reported to contribute to cell invasion and migration (Supplementary Table 6). Therefore, the number of cases carrying mutated genes implicated in cancer progression reaches a frequency of 39.4% (26 HR-Event3, 12 high-risk, 1 intermediate risk). The genes *ALK*, *COL1A1*, *FGFR1*, *GABRB2*, *HTRA1*, *MYLK2*, *PTEN* and *PTK2* showed drug-gene interactions (the Drug Gene Interaction database, DGIdb).

### Germline mutations

Given the low mutation rate and very early onset of neuroblastoma, we sought to identify germline mutations predisposing to aggressive neuroblastoma. Therefore, we screened germline sequence data for cancer predisposition genes [23] enriched in functional variants in 52 neuroblastoma patients (32 HR-Event3, 16 high-risk and 4 intermediate risk) compared to control populations (Table 1, Supplementary Figure 6, Supplementary Table 1 and 7). The top three genes most enriched in rare functional germline variants were *BARD1, CHEK2*, and *AXIN2* with 9 out of 11 mutations occurred in HR-Event3 patients (Supplementary Table 7).

**Table 1:** Results from the analysis of the enriched germline variants in 52 neuroblastoma patients

	Neuroblastoma		1000g-Control-Ita			1000g-Control-Eur			House-Control-Ita				
Gene	Patients with variants	Patients without variants	Controls with variants	Controls without variants	P-value	Controls with variants	Controls without variants	P-value	Controls with variants	Controls without variants	P-value	P-value combined population	Fold enrichment
BARD1*	4	100	0	107	**0.057**	0	396	**0.002**	0	106	**0.058**	**0.0004**	**25.0**
***AXIN2***	4	48	0	107	**0.011**	3	393	**0.004**	1	105	**0.041**	**0.0019**	**12.6**
*MC1R*	5	47	5	102	0.298	3	393	0.001	3	103	0.116	0.0056	5.8
***CHEK2***	3	49	0	107	**0.034**	3	393	**0.023**	0	106	**0.034**	**0.0078**	**12.5**
*SLC25A13*	2	50	0	107	0.106	2	394	0.068	0	106	0.107	0.0329	12.5
*CD96*	1	51	0	107	0.327	1	395	0.219	0	106	0.329	0.1513	12.3
*FH*	1	51	0	107	0.327	0	396	0.116	1	105	0.551	0.1513	12.3
*XRRC3*	1	51	0	107	0.327	1	395	0.219	0	106	0.329	0.1513	12.3
*APC*	2	50	2	105	0.597	7	389	0.281	1	105	0.252	0.2421	2.4
*RAD50*	1	51	0	107	0.327	2	394	0.310	2	104	1.000	0.337	3.0
*BRCA2*	1	51	1	106	0.549	3	393	0.391	1	105	0.551	0.3896	2.4
*SLX4*	1	51	0	107	0.327	5	391	0.525	0	106	0.329	0.3896	2.4
*FANCM*	1	51	1	106	0.549	3	393	0.391	3	103	1.000	0.4827	1.7
*PALB2*	1	51	5	102	0.665	2	394	0.310	2	104	1.000	0.5619	1.3

In bold germline variants significantly enriched in the neuroblastoma patients.

Enrichment of germline MC1R and SLC25A13 variants is considered not to be significant in neuroblastoma as no associaiton was found when using Italian controls.

* Data from 52 whole exome sequencing and 52 deep targeted sequencing of germilne DNA from neuroblastoma patients.

P-values calculated using 2-tailed Fisher's exact test.

## DISCUSSION

In attempt to better understand the neuroblastoma patho-biology at genomic level, 99 tumors were profiled by means of WES and DT-seq. Our study identifies novel candidate driver genes associated with clinically aggressive neuroblastoma (including HR-Event3 and high-risk phenotypes). As expected, most of these genes are infrequently mutated (from 4%-2%) but *in silico* analyses strongly support their functional involvement in neuroblastoma biology. This is in according to the recent literature suggesting that cancer genomes are composed of a few frequently mutated genes across patients but are dominated by a much larger number of infrequently mutated genes that contribute to disease initiation and progression [15].

Mutations in *CHD9* emerged as relatively recurrent in neuroblastoma (4%). *CHD9* (also known as CReMM, Chromatin Related Mesenchymal Modulator) is a chromatin remodeling protein. It is expressed during the differentiation of osteoprogenitors *in vivo* and *in vitro* and interacts with A/T-rich sites within promoters of key genes in osteoblast maturation [24]. Recent works dealing with the mechanisms of osteolytic metastasis in Stage IV neuroblastoma have highlighted that, similar to breast cancer and multiple myeloma, bone invasion by neuroblastoma cells is predominantly osteolytic and involves the activation of osteoclasts [25]. Here we report that a decreased gene expression of *CHD9* is associated with worst prognosis. In this scenario, loss of *CHD9* would lead to progression of neuroblastoma. Moreover, mouse insertional mutagenesis experiments support *CHD9* as a cancer driver gene (Candidate Cancer Gene Database). Indeed, clusters of insertional events of *CHD9* driving cancer progression have been reported in diverse tumors [26, 27] including malignant peripheral nerve sheath tumors [27] in which *CHD9* is deleted in 6.7% of cases. Intriguingly, the loss of the similar functional gene *CHD5* has been also linked to the progression of neuroblastoma tumors [28]. So far*, CHD9* mutations have not been reported in previous Next Generation Sequencing studies probably because in our study we used a restricted and homogeneous sub-set of aggressive neuroblastoma and because we profiled most of the tumors by DT-seq reaching coverage higher than 700x. In fact, the use of deep sequencing of a restricted panel of genes increases the sensitivity to detect mutations in known and potentially actionable genes.

Somatic mutations localized close to the well-known functional phosphorylation (Tyr576 and Tyr861) [11] sites were identified in *PTK2* (also known as FAK) in 2 cases. During development and in various tumours including neuroblastoma, FAK promotes cell motility, survival and proliferation through kinase-dependent and kinase-independent mechanisms [19, 20]. The anticancer compounds targeting FAK are currently in preclinical and clinical trials. Moreover, current literature suggests FAK inhibition for tumor suppression and prevention or delay of metastasis in neuroblastoma [11]. We think that the found somatic mutations could activate the FAK protein, which thus could be a promising therapeutic target for clinically aggressive neuroblastoma. Of note, non-silent somatic mutations were previously identified in *PTK2* in one primary and one relapsed neuroblastoma [9, 12].

Of particular interest was the identification of mutations in *HTRA1* encoding a member of the trypsin family of serine proteases, involved in the degradation of extracellular matrix proteins, important in cancer progression and invasion [21]. Several studies have indicated that the down regulation of *HTRA1* plays an important role in malignant progression of ovarian cancers [29] and melanoma [30]. A recent study has reported that the expression of HtrA1 protein is lower in advanced neuroblastomas [21]. In concordance with previous observations, we found variants that can act as loss-of-function mutations and thus can contribute to enhance invasion or metastasis of tumor cells. The mutation A180T in the exon 2 found in our study has been also identified in hepatocarcinoma and skin cutaneous melanoma whereas three missense mutations in exon 2 at 168, 166 and 167 position have been reported in hepatocarcinoma, pancreatic adenocarcinoma and lung adenocarcinoma, respectively. Therefore, the presence of a possible mutational hot spot in exon 2 can be hypothesized.

Two genes (*NAV1* and *NAV3*) belonging to the neuron navigator family were found mutated in 4 cases. *NAV3* is essential for the longitudinal growth of neurons involved in mechanosensation and mutant alleles can affect whole neuronal process bundles [31]. Accumulating evidence suggests that disruption of *NAV3* contributes progression of breast cancer [32], colorectal cancer [33], T-cell lymphoma [34] and nervous system tumors including neuroblastoma [22]. Based on current literature, we speculate that mutations in *NAV1* and *NAV3* could act as loss-of-function. This hypothesis is supported from the low gene expression levels of both genes found in advanced neuroblastomas.

Additional five mutated genes (*LRRC17*, *PXDN*, *FZD1*, *ARHGEF10L*, *ATRX)* were highly expressed in neuroblastoma and involved in cancer progression (Supplementary Table 6). Of particular interest is the gene *FZD1* because it promotes chemoresistance in neuroblastoma through activation of the Wnt/beta-catenin pathway [35].

There is growing interest in identifying disrupted pathways rather than single mutated genes in order to uncover biological systems perturbed in tumor cells [36]. Here we show that mutations in FA and RAC pathways (promoting metastasis and cancer progression) occur frequently in a subset of unfavourable neuroblastoma and confirm this finding in three independent sets of neuroblastoma samples. A recent study has suggested that dysfunction of FA pathway promotes bone marrow infiltration of stage 4 neuroblastoma [2]. Importantly, anticancer compounds targeting FA and RAC pathways can reduce growth, motility and viability of tumor cells in neuroblastoma [37]. In our study, combined results from single gene and pathway analysis indicated that 39.4% of patients (of which 66% were HR-Event3) carried mutations in genes involved in metastatic processes. We found 8 genes with potential druggability. We thus propose that inhibition of multiple steps of these processes could be of therapeutic interest and could yield better results in patients with aggressive neuroblastoma.

Neuroblastoma is an embryonal tumor, which arises in the fetus or very early in life and is likely to be less influenced by environmental factors than adult malignancies. We have already demonstrated by using genome-wide association studies that common variants are associated with risk of neuroblastoma [4-7]. We thus speculated that also rare germline variants could have a role in the etiology of this pediatric malignancy. We identified significant enrichment of rare germline variants in *BARD1*, *CHEK2* and *AXIN2.* Neuroblastoma-predisposing mutations in *BARD1* and *CHEK2* have been recently reported [9]. *BARD1* is a known neuroblastoma susceptibility locus [4] but little is known about the frequencies of rare variants. Here, we found the same, rare, loss-of-function mutation (BARD1 p.Arg641*) that has been identified by Pugh et al. [9]. *AXIN2* is involved in the regulation of Wnt/β-catenin and neural crest differentiation pathways, and its mutations have been reported in association with gastrointestinal cancers [38]. Interestingly, we already demonstrated that Wnt/β-catenin signaling is activated in high-risk neuroblastoma without *MYCN* amplification [39]. Our data suggest that rare germline genetic variants might cooperate to determine the rapidly progressive nature of clinically aggressive neuroblastomas.

In this study we identified diverse genes mutated at relatively low frequency in high-risk and HR-event3 neuroblastoma. It is intriguing that chromatin modeler *CHD9* is the most promising cancer driver. In according to metastatic propensity of studied tumors, most of the genes have been reported to play key roles in cell migration and motility suggesting that the functional impact, or the role in suppressing or promoting a tumor, of a single mutation is not static but depends on cell state and the presence of other mutations and could have effects on multiple cellular processes. Indeed, we also demonstrated FA and RAC pathways as frequently mutated in this sub-set of aggressive neuroblastoma. Our finding raises a new challenge in the treatment of cancer that involves the use of a selection of therapies based on different genetic alterations in individual tumors. We believe that our understanding of cancer biology through the lens of pathway-level implication is nascent, but it holds the potential to transform our thinking on disease etiology and treatment.

## MATERIALS AND METHODS

### Somatic mutation identification

#### Samples collection

Neuroblastoma tumor DNA (primary tumors) and matched germline DNA (from peripheral blood) were obtained from the IRCCS AOU San Martino-IST and Hospital Sant Joan de Déu, Esplugues de Llobregat. Primary tumor samples were verified to have > 75% viable tumor cell content by histopathology assessment. The human-derived neuroblastoma cell lines were purchased from commercial sources and the nucleic acids (mRNA and DNA) were extracted from each of the original purchased cryotube after keeping the cells in culture for only 5-7 passages from thawing. All cell lines were screened for the presence of mycoplasma. The patients were classified in low, intermediate and high risk groups based on International Neuroblastoma Risk Groups criteria [40]. We selected a subgroup of patients with rapid progression disease and named “HR-Event3” which included high-risk individuals with any adverse event (tumor progression, relapse or death) within 36 months from diagnosis. Our discovery set of 17 HR-Event3 neuroblastomas was composed of 15 (88%) high-risk individuals with an adverse event within 18 months from diagnosis. Therefore the tumors subjected to whole exome sequencing were highly aggressive. The human-derived neuroblastoma cell lines were classified as high-risk. Informed consent for research use was obtained from all subjects and/or parents and study approval was obtained from Ethics Committee of the Medical University of Naples. Detailed clinical information of neuroblastoma patients and biological information of tumor samples are provided in Supplementary Table 1.

#### High throughput sequencing

Exome regions were captured and enriched with the Agilent SureSelect Target Enrichment System 50Mb (Agilent Technologies, Santa Clara, CA) according to the manufacturers' protocol. Whole Exome Sequencing was performed on an Illumina HiSeq 2000 (Illumina Inc., San Diego, CA) yielding 100bp paired-end reads with a mean depth of 110x (Supplementary Table 2a). Deep Targeted sequencing (DT-seq) was designed considering the coding regions (plus 10bp at 5′ and 3′ ends) of the 134 candidate cancer driver genes. The DNA was captured using the SeqCap EZ Library SR (Roche NimbleGen, Madison, WI). Captured DNAs were subjected to massively parallel sequencing using an Illumina HiSeq 1000 obtaining 100bp paired-end reads with a mean depth of 733x (Supplementary Table 2b).

#### Sequencing data processing and mutation calling

Burrows-Wheeler Aligner [41] was used to map sequencing reads *versus* the human reference genome, assembly GRCh37/hg19. Alignment files for tumor and control tissue pairs, were piled up with Samtools [42] and variants (Single Nucleotide Variations and Short Insertions/Deletions) were called using VarScan2 [43] and annotated with Annovar [44]. As explained in detail in Supplementary information, raw variant calls were strongly filtered to get a high confidence set of functionally relevant somatic changes that were manually curated and visually inspected with the IGV - Integrated Genome Viewer.

#### Somatic signature profiling

Somatic single-nucleotide variations (SNVs), divided into 96 groups defined by substitution class and sequence context (adjacent bases at 3′ and 5′), were used to draw a somatic signature profile. To compare and validate our results, we drew somatic signatures for the lists of somatic mutations from recent next generation sequencing-based screenings on primary neuroblastomas: Molenaar et al. [8], Pugh et al. [9] and Sausen et al. [10].

#### Prioritization of driver mutations

We used the Cancer-specific High-throughput Annotation of Somatic Mutations (CHASM) [45] tool to distinguish passenger variation events from driver ones without considering the mutation recurrence across our cohort of tumors. The program predictions are based on the probability that a somatic missense variant can increase the fitness of cancer cells. The Variant Effect Scoring Tool (VEST) [46] was used to identify variants that affect the molecular function of the protein and prioritize them on the basis of the likelihood of their involvement in human disease. Details on filtering steps and candidate cancer drivers inclusion criteria are provided in Supplementary information.

#### Gene expression analysis

Normalized gene expression array data of two independent sets of neuroblastoma patients were downloaded from the website “R2: Genomics Analysis and Visualization Platform” and named “Affymetrix data” (GEO ID: GSE16476) and “Agilent data” (GEO ID: GSE49710); see Supplementary information. The comparison of gene expression profiles (Log2 transformed) among low-risk, high-risk and HR-Event3 patients was performed with “R2” using the following parameters: 1) *T*-test to assess the statistical significance; 2) FDR to correct for multiple tests. Enriched gene sets were supported by significance statistical analysis with hypergeometric test.

#### Identification of significantly altered pathways

We ranked the list of mutated genes found by whole exome sequencing (WES) and DT-seq methods according to the score of pathogenicity assigned by CHASM algorithm. Then we applied Gene Set Enrichment Analysis (GSEA) that assesses if a pre-defined set of genes has more high-ranking genes than would be expected by chance. The same analysis was also conducted on the list of mutated genes showed in supplementary data of the previously mentioned works [8-10]. We used the canonical pathways defined by the KEGG database.

#### Sanger sequencing validation of somatic mutations

A total of 70 somatic SNVs and indels identified by next-generation sequencing (WES and DT-seq) were verified by Sanger sequencing. All somatic variants, except for 2 SNVs, were successfully validated. Primers sequences are available upon request.

### Germline mutation identification

#### Samples collection and data analysis

We used WES data from blood-derived DNA samples of the 17 neuroblastoma patients used for the somatic mutation identification, 28 additional neuroblastoma patients (obtained from the National Cancer Research Institute of Genoa), and 7 neuroblastoma patients (Supplementary Table 1) reported in the paper of Sausen et al. [10] downloaded from European Genome-Phenome Archive (EGAC00001000085).

#### Filtering of germline mutations

Germline variants were filtered step by step to identify the potentially interesting candidates applying a similar approach successfully used by Wang L et al. [47]. The detailed workflow is described in Supplementary information. The subset of functionally relevant variants in known cancer predisposition genes [23] was filtered from common polymorphisms (reported in dbSNP135 given that common SNPs are unlikely to be disease causative), then mutation calls with a VEST 3.2 *p*-value < 0.10 (predicted to be pathogenic) were kept for further analysis. For each gene in the above list, we calculated the fold of enrichment in germline variants in neuroblastoma patients and compared to that of control cohorts (106 controls from in-house exome data, 107 Italian controls from 1000 Genome Project, 396 European controls from 1000 Genome Project). The potentially interesting and candidate neuroblastoma-predisposing genes were then selected on the basis of the significant P values calculated with the Fisher's exact test.

#### Sanger sequencing validation of germline mutations

A total of 15 germline SNVs identified by next-generation sequencing were verified by Sanger sequencing. All variants were successfully confirmed. Primers sequences are available upon request.

## SUPPLEMENTARY MATERIAL FIGURES AND TABLES
















