# Supplementary material for: Exome and deep sequencing of clinically aggressive neuroblastoma reveal somatic mutations that affect key pathways involved in cancer progression
Source: Oncotarget. 2016 Mar 18;7(16):21840–52. doi: 10.18632/oncotarget.8187 (PMC5008327; doi:10.18632/oncotarget.8187)
Supplement: Supplementary file 1 [file oncotarget-07-21840-s001.pdf]

Exome and deep sequencing of clinically aggressive neuroblastoma reveal somatic mutations that affect key pathways involved in cancer progression

Supplementary Material

1. Somatic mutation identification

Next generation sequencing data processing and mutation calling

Illumina paired-end reads were mapped versus the reference genome (GRCh37/hg19 downloaded from UCSC Genome Browser) using the Burrows-Wheeler Aligner [1] algorithm; we disallowed indels within 15bp towards the ends of the read and trimmed down bases with quality less than 10 at 3' to reduce false positives due to sequencing errors. Alignment information, stored in Binary Alignment-Map files, for tumor and control tissue pairs, was piled up with SamTools [2] and variants were called using VarScan2 [3] with default parameters (minimum coverage to call a variant = 8, minimum coverage to call Somatic = 8 in normal, and = 6 in tumor). Genetic variants identified were annotated for their presence in several databases such as dbSNP-135, 1000 Genome Project, COSMIC [4] (version 68) and ESP6500 using ANNOVAR [5]. Annotated variants were strongly filtered to get a high confidence set of somatic changes. We kept only the variants: *i*) with the p-value < 0.01 (Fisher Exact Test implemented in VarScan2); *ii*) not registered in either dbSNP135 or 1000 Genome Project; *iii*) not located in duplicated genomic regions.
Putative somatic variants were then split on the basis of their functional class as annotated by ANNOVAR. Off-target variants, annotated as intergenic, intronic, upstream or downstream (variants up to 1-Kb away from the transcription start or end site) were discarded. Variants with a strand bias over the 90% were set aside to reduce false positive calls. The set of mutations obtained was manually curated and visually inspected with the IGV - Integrated Genome Viewer [6].
To detect probable somatic mutations of 17 HR-Event3 tumors and 17 cell lines that not have matched germline DNA, we excluded all variants reported in all dbSNP builds, 1000 Genome Project, ESP6500 database and 106 exomes from in-house Italian controls.
DT-Seq (Deep Targeted Sequencing) raw variant calls were filtered with the same parameters used for WES (Whole Exome Sequencing) data, except for the somatic p-value from VarScan2 (less than 0.05; see above), a minimum total depth of 10, and an altered allele frequency in the normal tissue under 3%. After filtering steps described above, of variant allele frequencies in tumor were >= 20% and >=17% for the exome and for the DT-Seq, respectively.

In-silico validation

All steps of our analyses were followed by consistency checks of the results. As yardsticks we used the list of somatic mutations of recent next generation sequencing-based screenings on primary neuroblastomas: Molenaar et al. [7], Pugh et al. [8] and Sausen et al. [9]. We searched for the presence of our mutated genes in their datasets (Supplementary Table 3a,b), compared mean values of somatic variants per sample (Supplementary Table 2c). A somatic signature profile was built on these data (Supplementary Figure 2) and compared to ours (Figure 1c,d). Cancer driver analysis was run on the above mentioned lists of variants in order to obtain sets of somatic driver mutations that were further investigated by KEGG Pathway analysis (see below) and compared to our data.

Prioritization of driver mutations

Neuroblastoma is known to harbour few somatic mutations [7-9]. Therefore, to identify rare driver mutations, we decided to use algorithms that do not take into account the recurrence of mutations in

a cohort of cancer patients. We used Cancer-Related Analysis of Variants Toolkit (CRAVAT [10]) version 3.2, which implements the Cancer-specific High-throughput Annotation of Somatic Mutations (CHASM [11]) tool, to distinguish passenger variation events from driver ones. In brief CHASM implements a random forest machine learning method trained on a positive class of driver events curated from the COSMIC database and a negative set of *in silico* generated passengers consistent with passenger base substitution frequencies estimated for a specific tumor type. The program predictions are based on the probability that a somatic missense variant can increase the fitness of cancer cells. The Variant Effect Scoring Tool VEST [12], a supervised machine learning-based classifier implemented in CRAVAT, was used to assess the functional effect of variants and prioritize them on the basis of the likelihood of their involvement in human disease. To select cancer driver events, we maintained WES somatic variants on the basis of the following criteria: *i*) genes with p<0.05, which was calculated by CHASM, *ii*) genes with p<0.05 which was calculated by VEST. Moreover, we included all splicing and stop-gain somatic events and only Insertion/Deletion (indel) variants predicted to be damaging by SIFT [13]. This approach allowed us to identify functional significance of frequent and rare mutations. This process readily included well-studied cancer drivers such as *ALK*, *ERBB3*, *PTEN*, *PTK2, FGFR1* and excluded many common passenger mutations, for example, *TTN* and the genes encoding mucins, cytoskeletal dyneins and olfactory receptors. Using this approach on WES data, we selected a total of 125 candidate driver genes (highlighted in grey in Supplementary Table 3a), which were subjected to DT-seq in 82 neuroblastomas. We also included 9 other candidate genes manually selected according to the following criteria: *i*) genes (*ATRX*, *PTPN11*, *NRAS*, *ARID1A*, *MYCN*) found significantly mutated in previous studies [7-9] and registered in Cancer Gene Census database; *ii)* *TENM3* and *ADCY10* found mutated in Molenaar et al. [7] and functionally similar to the genes *TENM4*, *ADCY6*, *ADCY3* that were mutated in our discovery set; *iii*) *BARD1* since it is a known neuroblastoma susceptibility gene [14] and found mutated in our discovery set; *iv*) *RET* as functionally relevant gene in neuroblastoma according to not published data of our laboratory and registered in Cancer Gene Census database.

To identify cancer driver genes in combined cohorts from WES and DT-seq, we recalculated the p-values for all mutations and selected candidate cancer driver genes by applying the following filtering criteria: *i*) False Discovered Rate (FDR) ≤0.25 for CHASM or FDR≤0.1 for VEST; *ii*) genes mutated in at least two cases. After this analysis, 22 genes resulted to be significantly mutated. The combined p-value for each gene was calculated based on Stouffer's Z-score method [15].

Gene expression analysis

Normalized gene expression array data of two independent sets of neuroblastoma patients were downloaded from the website "R2: Genomics Analysis and Visualization Platform (http://r2.amc.nl)": *i*) "Affymetrix data" composed of 88 samples (Affymetrix Human Genome U133 Plus 2.0 Array, GEO ID: GSE16476); *ii*) "Agilent data" composed of 498 samples (Agilent-020382 Human Custom Microarray 44k, GEO ID: GSE49710). Log2 transformed data were used for both gene expression datasets. The comparison of gene expression profiles among low-risk, high-risk and HR-Event3 patients was performed by the R2: Genomics Analysis and Visualization Platform using the following parameters: 1) T-test to assess the statistical significance; 2) FDR to correct for multiple tests. Enriched gene sets were supported by significance statistical analysis with hypergeometric test. As the elevate number of patients in Agilent dataset, we were able to use two different categories of HR-Event characterized patients: high-risk individuals with any adverse event within *i*) 36 (HR-Event3) and *ii*) 18 (HR-Event1.5) months from diagnosis (Supplementary Figure 4 and Supplementary Table 5).

To identify the relative expression of genes in neuroblastomas, the 25th percentile value of the expression of the significantly mutated genes (Figure 2A) was computed. This analysis was conducted on the Affymetrix and Agilent microarray data. The median percentiles for *NEB*,

COL6A6, *GABRB2*, *SIRPB2*, *DTHD1*, *PCDHGB1*, *MYH7* (less than the 25th percentiles in both datasets) suggest low expression in neuroblastoma tumors (Supplementary Figure 3Aand 3B) Gene expression comparison across 491 types of cancers was performed by Genevisible webtool (Supplementary Figure 3C and 3D).

URLs:

Cosmic: cancer.sanger.ac.uk/cosmic. SIFT: sift.jcvi.org. PolyPhen: genetics.bwh.harvard.edu. Mutation Assessor: mutationassessor.org. Mutation Taster: www.mutationtaster.org. KEGG Pathway Database: www.genome.jp/kegg/pathway. cBioPortal: www.cbioportal.org. R2: Genomics Analysis and Visualization Platform http://r2.amc.nl. 1000 Genomes Project: http://www.1000genomes.org/. PhosphoSitePlus: http://www.phosphosite.org/ Candidate Cancer Gene Database (CCGD): http://ccgd-starrlab.oit.umn.edu/. The Cancer Genome Atlas: http://cancergenome.nih.gov/. Genevisible: http://genevisible.com/. the Drug Gene Interaction database, DGIdb: http://dgidb.genome.wustl.edu/.

References

1. Li H and Durbin R. Fast and accurate short read alignment with Burrows-Wheeler transform. Bioinformatics. 2009; 25(14):1754-1760.
2. Li H, Handsaker B, Wysoker A, Fennell T, Ruan J, Homer N, Marth G, Abecasis G, Durbin R and Genome Project Data Processing S. The Sequence Alignment/Map format and SAMtools. Bioinformatics. 2009; 25(16):2078-2079.
3. Koboldt DC, Zhang Q, Larson DE, Shen D, McLellan MD, Lin L, Miller CA, Mardis ER, Ding L and Wilson RK. VarScan 2: somatic mutation and copy number alteration discovery in cancer by exome sequencing. Genome research. 2012; 22(3):568-576.
4. Forbes SA, Beare D, Gunasekaran P, Leung K, Bindal N, Boutselakis H, Ding M, Bamford S, Cole C, Ward S, Kok CY, Jia M, De T, Teague JW, Stratton MR, McDermott U, et al. COSMIC: exploring the world's knowledge of somatic mutations in human cancer. Nucleic acids research. 2015; 43(Database issue):D805-811.
5. Wang K, Li M and Hakonarson H. ANNOVAR: functional annotation of genetic variants from high-throughput sequencing data. Nucleic acids research. 2010; 38(16):e164.
6. Robinson JT, Thorvaldsdottir H, Winckler W, Guttman M, Lander ES, Getz G and Mesirov JP. Integrative genomics viewer. Nature biotechnology. 2011; 29(1):24-26.
7. Molenaar JJ, Koster J, Zwijnenburg DA, van Sluis P, Valentijn LJ, van der Ploeg I, Hamdi M, van Nes J, Westerman BA, van Arkel J, Ebus ME, Haneveld F, Lakeman A, Schild L, Molenaar P, Stroeken P, et al. Sequencing of neuroblastoma identifies chromothripsis and defects in neuritogenesis genes. Nature. 2012; 483(7391):589-593.
8. Pugh TJ, Morozova O, Attiyeh EF, Asgharzadeh S, Wei JS, Auclair D, Carter SL, Cibulskis K, Hanna M, Kiezun A, Kim J, Lawrence MS, Lichenstein L, McKenna A, Pedamallu CS, Ramos AH, et al. The genetic landscape of high-risk neuroblastoma. Nature genetics. 2013; 45(3):279-284.
9. Sausen M, Leary RJ, Jones S, Wu J, Reynolds CP, Liu X, Blackford A, Parmigiani G, Diaz LA, Jr., Papadopoulos N, Vogelstein B, Kinzler KW, Velculescu VE and Hogarty MD. Integrated genomic analyses identify ARID1A and ARID1B alterations in the childhood cancer neuroblastoma. Nature genetics. 2013; 45(1):12-17.
10. Douville C, Carter H, Kim R, Niknafs N, Diekhans M, Stenson PD, Cooper DN, Ryan M and Karchin R. CRAVAT: cancer-related analysis of variants toolkit. Bioinformatics. 2013; 29(5):647-648.
11. Carter H, Chen S, Isik L, Tyekucheva S, Velculescu VE, Kinzler KW, Vogelstein B and Karchin R. Cancer-specific high-throughput annotation of somatic mutations: computational prediction of driver missense mutations. Cancer research. 2009; 69(16):6660-6667.
12. Carter H, Douville C, Stenson PD, Cooper DN and Karchin R. Identifying Mendelian disease genes with the variant effect scoring tool. BMC genomics. 2013; 14 Suppl 3:S3.
13. Ng PC and Henikoff S. SIFT: Predicting amino acid changes that affect protein function. Nucleic acids research. 2003; 31(13):3812-3814.
14. Capasso M, Devoto M, Hou C, Asgharzadeh S, Glessner JT, Attiyeh EF, Mosse YP, Kim C, Diskin SJ, Cole KA, Bosse K, Diamond M, Laudenslager M, Winter C, Bradfield JP, Scott RH, et al. Common variations in BARD1 influence susceptibility to high-risk neuroblastoma. Nature genetics. 2009; 41(6):718-723.
15. Whitlock MC. Combining probability from independent tests: the weighted Z-method is superior to Fisher's approach. Journal of evolutionary biology. 2005; 18(5):1368-1373.

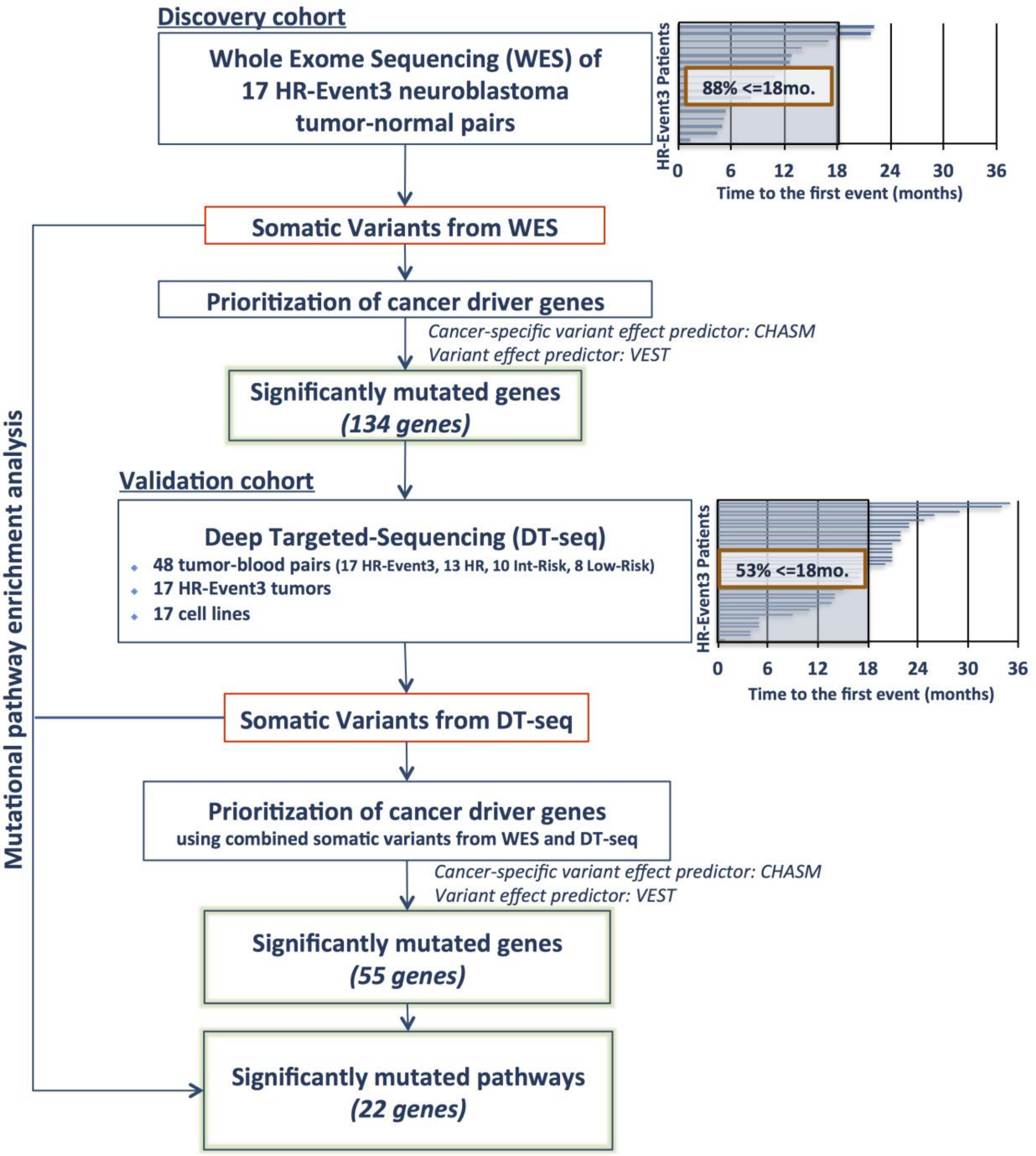

Supplementary Figure 1

Research strategy to identify somatically mutated genes and pathways. Whole exome sequencing was performed on 17 HR-Event3 neuroblastoma and matched control tissue samples, whereas deep targeted sequencing was performed on a total of 82 samples. The significantly mutated genes and those manually selected (see Supplementary information) were included in the targeted gene panels. Mutated genes and pathways were evaluated combining data obtained from both cohorts (discovery and validation). The cancer driver mutations were prioritized by CHASM and VEST algorithms that do not consider recurrence of the variants and thus are suitable to detect

infrequently mutated genes for a pediatric cancer like neuroblastoma, which is known to harbor few somatic mutations. The plots on the right report the time to first event from the date of diagnosis for each patient.

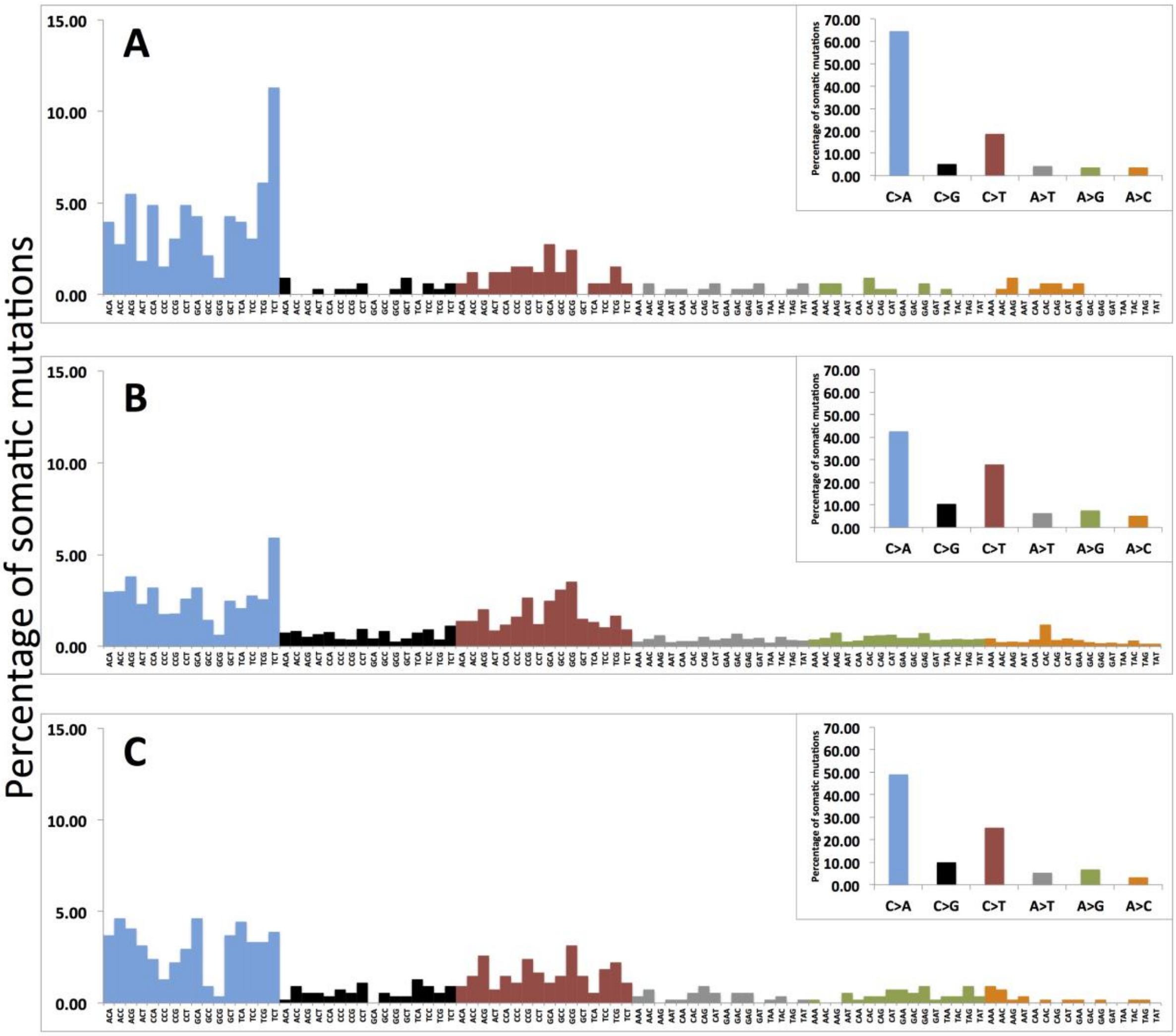

Supplementary Figure 2

Signature of somatic mutations in neuroblastoma. Each panel shows the detailed spectrum of somatic point mutations, for each group of trinucleotides, found in neuroblastoma. The Y axis reports the frequency of nucleotide substitutions, while X axis shows the trinucleotides (the variant site plus the bases at 5' and 3') in which the somatic changes occur. The top right boxes indicate the frequency of the six types of base substitutions caused by somatic mutations in each data set. **A,** Somatic mutations published in Sausen et al. [9], **B,** in Pugh et al. [8], **C,** in Molenaar et al. [7].

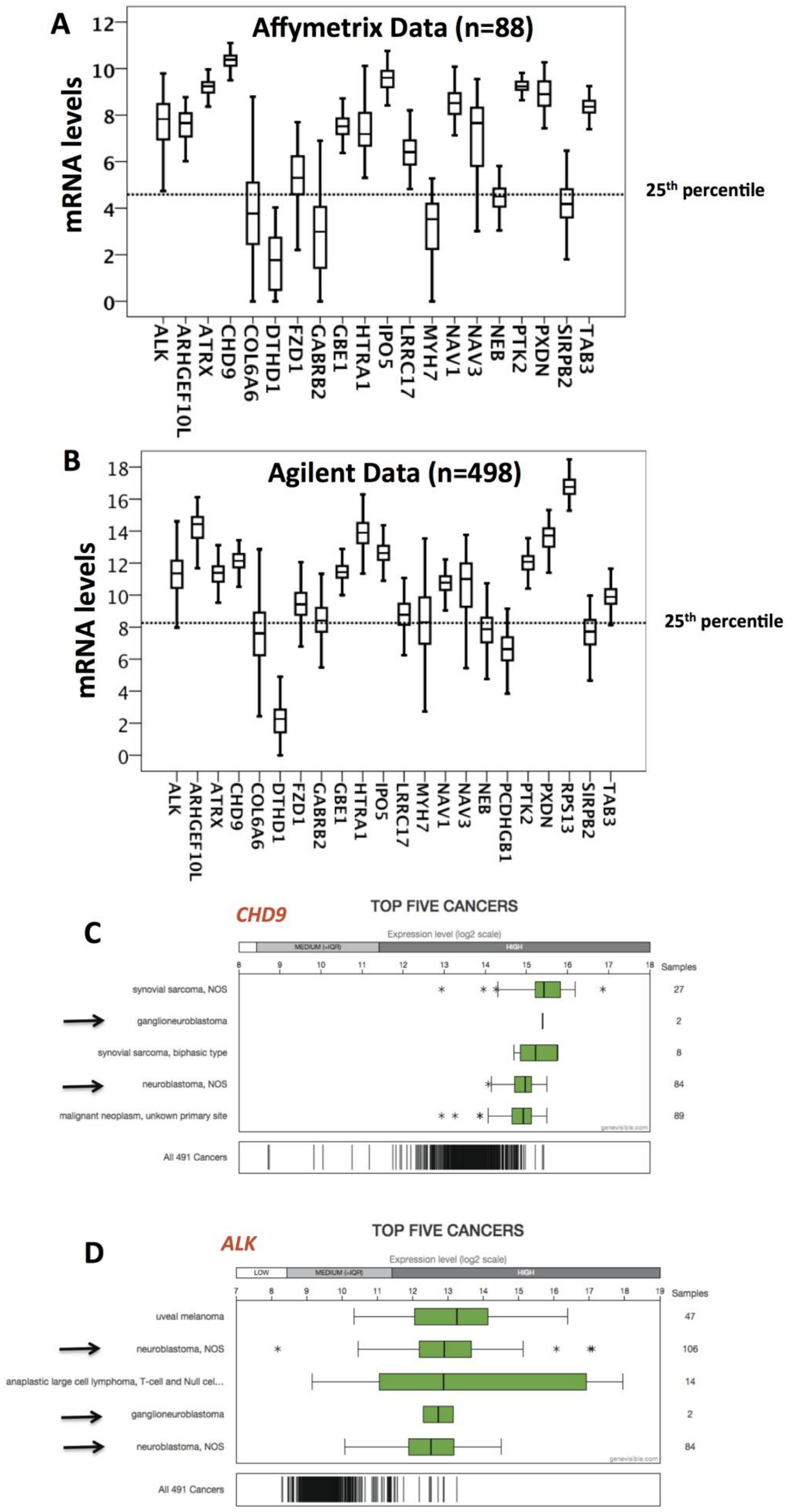
A
Affymetrix Data (n=88)
mRNA levels
25th percentile
ALK
ARHGEF10L
ATRX
CHD9
COL6A6
DTHD1
FZD1
GABRB2
GBE1
HTRA1
IPO5
LRRC17
MYH7
NAV1
NAV3
NEB
PTK2
PXDN
SIRPB2
TAB3
B
Agilent Data (n=498)
mRNA levels
25th percentile
ALK
ARHGEF10L
ATRX
CHD9
COL6A6
DTHD1
FZD1
GABRB2
GBE1
HTRA1
IPO5
LRRC17
MYH7
NAV1
NAV3
NEB
PCDHGB1
PTK2
PXDN
RPS13
SIRPB2
TAB3
C
CHD9
TOP FIVE CANCERS
Expression level (log2 scale)
MEDIUM (-IQR)
HIGH
Samples
synovial sarcoma, NOS
27
ganglioneuroblastoma
2
synovial sarcoma, biphasic type
8
neuroblastoma, NOS
84
malignant neoplasm, unkown primary site
89
All 491 Cancers
D
ALK
TOP FIVE CANCERS
Expression level (log2 scale)
LOW
MEDIUM (-IQR)
HIGH
Samples
uveal melanoma
47
neuroblastoma, NOS
106
anaplastic large cell lymphoma, T-cell and Null cel...
14
ganglioneuroblastoma
2
neuroblastoma, NOS
84
All 491 Cancers

Supplementary Figure 3

Comparison of expression levels of significantly mutated genes in neuroblastoma. The distribution of expression values of each gene in **A,** 88 primary neuroblastoma tumors profiled by Affymetrix Human Genome U133 Plus 2.0 Array (GEO ID: GSE16476) and **B,** 498 primary neuroblastoma tumors profiled by Agilent 44K expression microarrays (GEO ID: GSE49710). *PCDHGB1* and *RPS13* were not included in Affymetrix array. **C,** Expression of *CHD9* (212616_at) and **D,** *ALK* (208212_s_at) across 491 types of cancers tested by Genevisible web tool (http://genevisible.com/). These two genes seem to be specifically expressed in neuroblastoma.

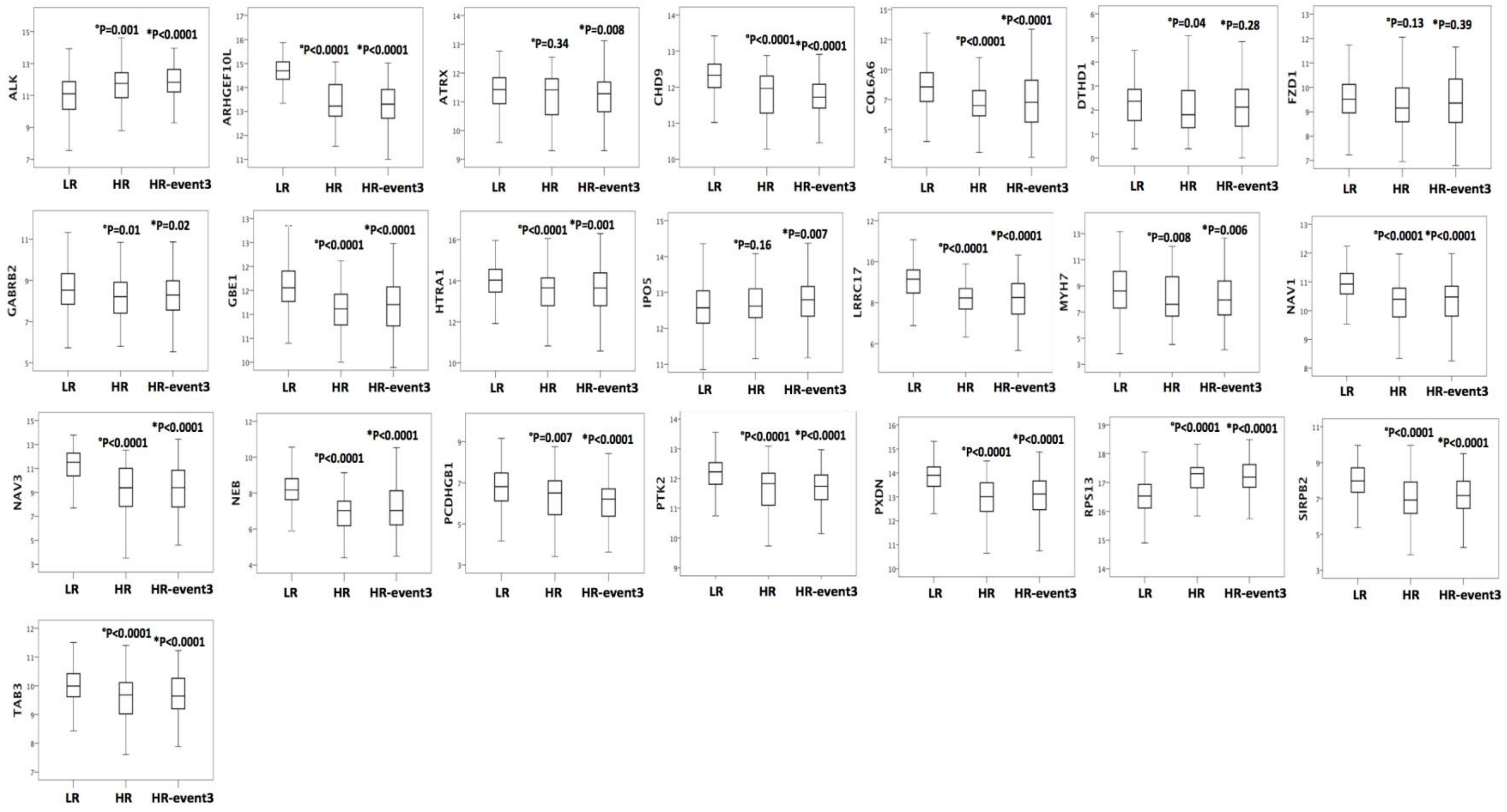

Supplementary Figure 4A

Gene expression profiles of significantly mutated genes in 498 neuroblastomas: 322 low risk (LR), 63 high risk (HR) and 113 HR-Event3 (*high-risk individuals with any adverse event within* ***36 months from diagnosis***).

Y axis indicates the mRNA levels for each gene. GEO ID: GSE49710.

*P-value calculated by Manny-Whitney test comparing LR and HR tumors °P-value calculated by Manny-Whitney test comparing HR and HR-Event3 tumors

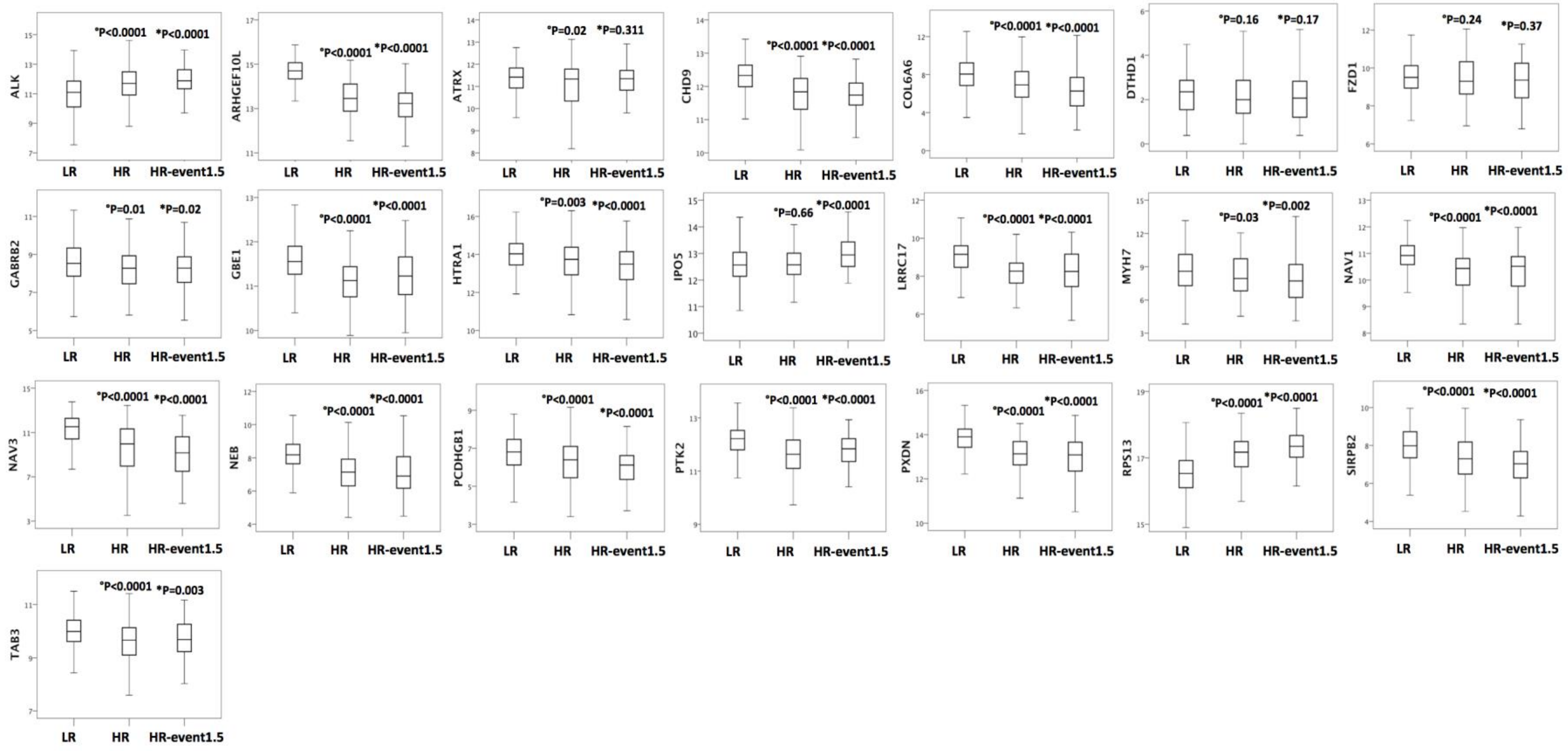

Supplementary Figure 4B

Gene expression profiles of significantly mutated genes in 498 neuroblastomas: 322 low risk (LR), 102 high risk (HR) and 74 HR-Event1.5 (*high-risk individuals with any adverse event within* ***18 months from diagnosis***).

Y axis indicates the mRNA levels for each gene. GEO ID: GSE49710.

*P-value calculated by Manny-Whitney test comparing LR and HR tumors °P-value calculated by Manny-Whitney test comparing HR and HR-Event1.5 tumors

Affymetrix Data (n=88)

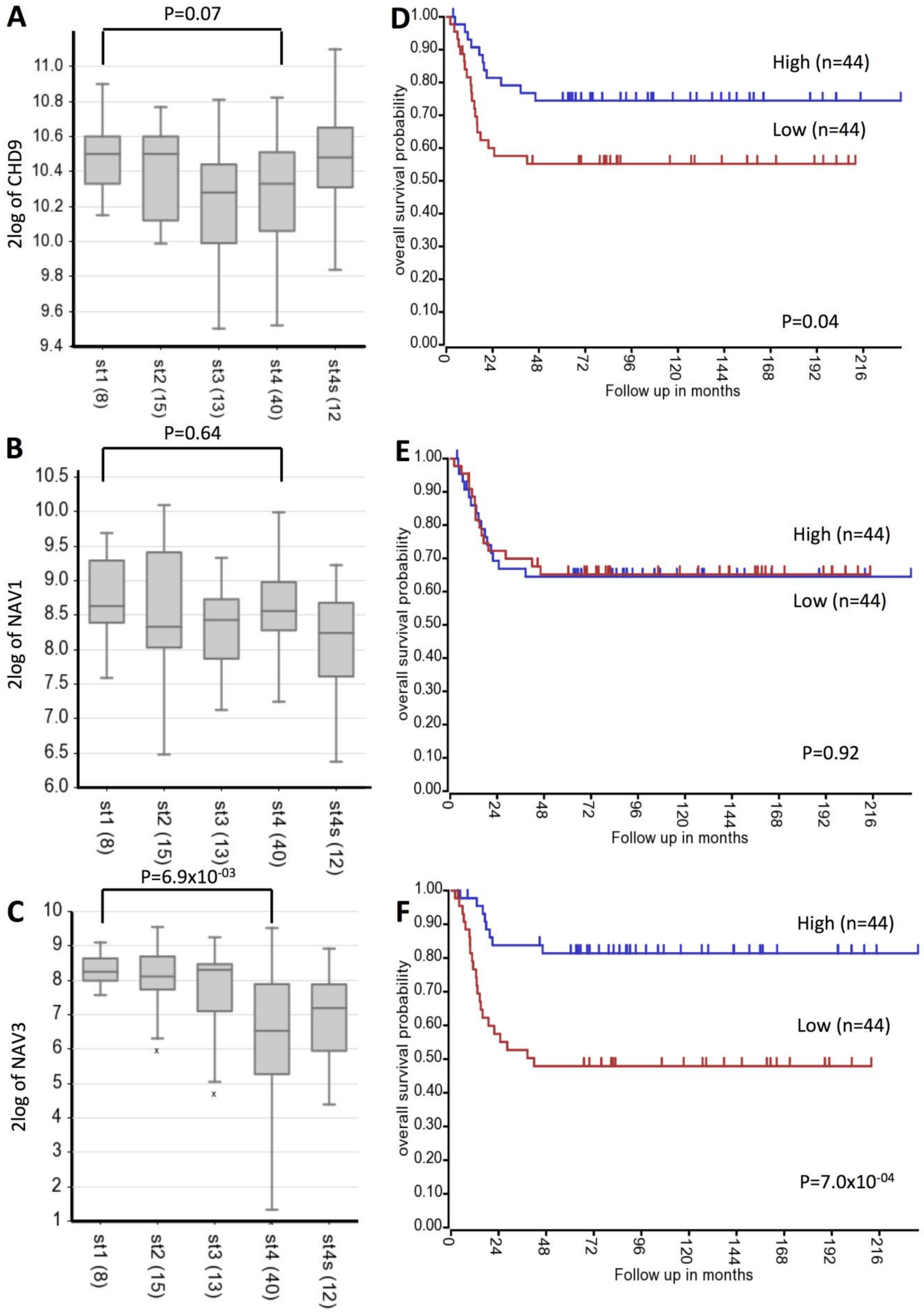

Supplementary Figure 5

CHD9, NAV1 and NAV3 expression levels and survival rates. Low *CHD9* and *NAV3* expression is associated with negative prognosis and metastatic neuroblastoma stage. **A-B-C,** Changes in expression for *CHD9, NAV1* and *NAV3* respectively, in advanced-stage neuroblastoma using published array data (R2 bioinformatics tool). Data are shown for International Neuroblastoma Staging System, stages 1–4 and 4s. The number of tumors is indicated in parentheses. **D-E-F,** Kaplan-Meier analysis is shown, with individuals grouped by median of expression of *CHD9, NAV1* and *NAV3*, respectively. Log-rank P values are shown.

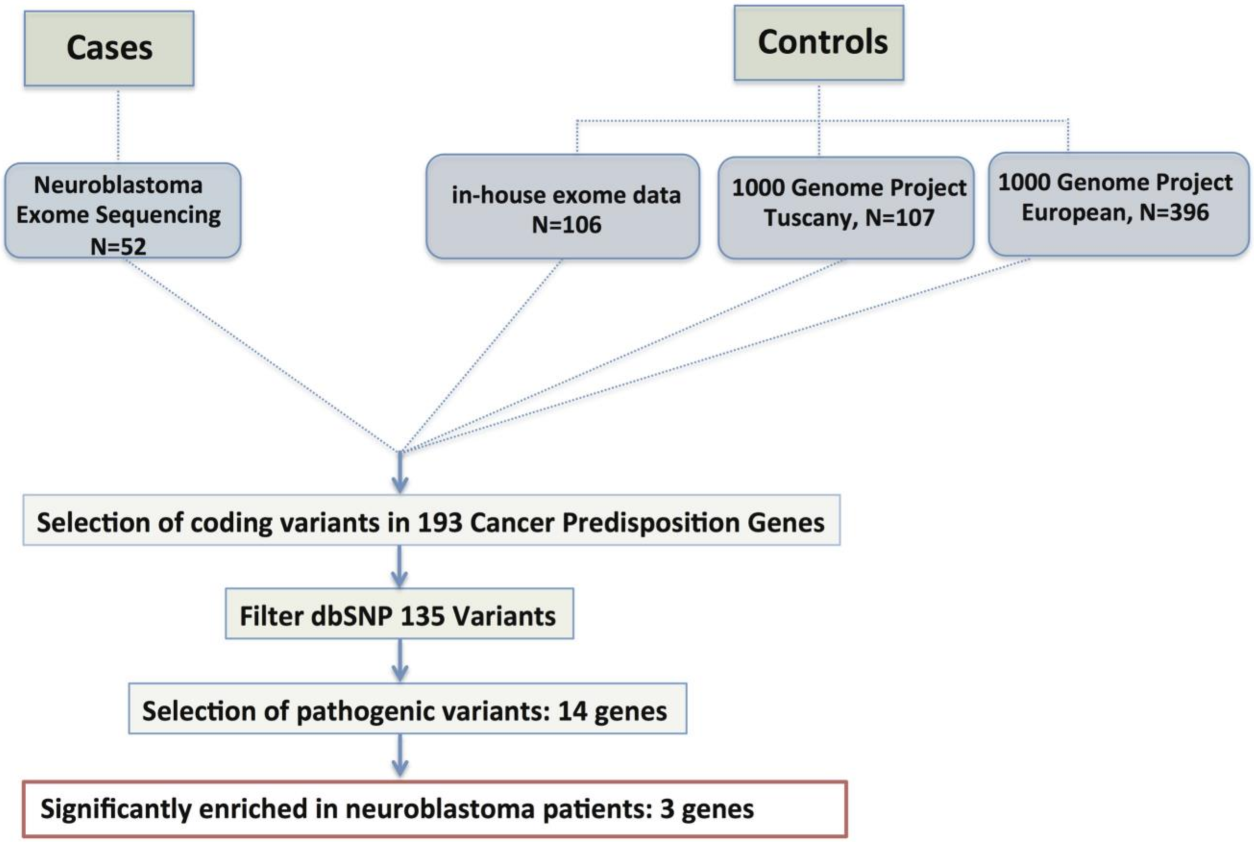

Supplementary Figure 6

Research strategy to identify neuroblastoma-predisposing variants. Germline variants were filtered step by step to pick up the potentially interesting cancer genes candidates. First, the non-silent variants including missense, nonsense, indels, and splice-site variants were selected. Second, novel variants that have not been reported in dbSNP database (dbSNP135) were selected. Then, variants predicted to be pathogenic (VEST tool) with a p-value < 0.10 were selected. After that, for 14 genes, the fold of enrichment of the germline variants in neuroblastoma patients were calculated comparing the frequency in the control cohort and performed Fisher's exact test to calculate the P values. Finally, potentially interesting genes based on significant P-values (<0.05) were selected.
